# Mitochondria: Redox Metabolism and Dysfunction

**DOI:** 10.1155/2012/896751

**Published:** 2012-04-24

**Authors:** Jia Kang, Shazib Pervaiz

**Affiliations:** ^1^ROS, Apoptosis and Cancer Biology Laboratory, Department of Physiology, Yong Loo Lin School of Medicine, National University of Singapore, Singapore 117597; ^2^NUS Graduate School for Integrative Sciences and Engineering, National University of Singapore, Singapore 117597; ^3^Cancer and Stem Cell Biology Program, Duke-NUS Graduate Medical School, Singapore 169857; ^4^Cell and Systems Biology (CSB), Singapore-MIT Alliance, Singapore 637460

## Abstract

Mitochondria are the main intracellular location for fuel generation; however, they are not just power plants but involved in a range of other intracellular functions including regulation of redox homeostasis and cell fate. Dysfunction of mitochondria will result in oxidative stress which is one of the underlying causal factors for a variety of diseases including neurodegenerative diseases, diabetes, cardiovascular diseases, and cancer. In this paper, generation of reactive oxygen/nitrogen species (ROS/RNS) in the mitochondria, redox regulatory roles of certain mitochondrial proteins, and the impact on cell fate will be discussed. The current state of our understanding in mitochondrial dysfunction in pathological states and how we could target them for therapeutic purpose will also be briefly reviewed.

## 1. Introduction

Mitochondria are the main intracellular location for generating adenosine triphosphate (ATP), the fuel for cell's metabolic needs, and therefore are referred to as the power plants of the cell. Energy is stored in the form of phosphate bond and is released when ATP is hydrolyzed to adenosine diphosphate (ADP) to meet the requirement of a number of energy demanding cellular processes. ATP is generated through cellular respiration, including a set of chemical reactions named as the tricarboxylic acid (TCA) cycle and oxidative phosphorylation that take place in mitochondria; therefore, mitochondria play a crucial regulatory role in cellular metabolism [1]. However, mitochondria are far more than just power suppliers. They are also involved in many other cellular functions, including calcium signaling [2], heme [3] and steroid synthesis [4], regulation of membrane potential [1], proliferation [5] or apoptosis [6], and redox homeostasis maintenance [7], to name just a few. Mitochondria are the major sites for free radical species production, including both reactive oxygen species (ROS) and reactive nitrogen species (RNS). On one hand, free radical species are indispensable for proper cell signaling; on the other hand, excessive generation of ROS results in cell/tissue injury and death. In this paper, the underlying mechanisms for generation of free radical species in the mitochondria and how some mitochondrial proteins act as redox regulators will be of prime focus. In the past decade, more and more pieces of evidence are surfacing to point the role of ROS as critical mediators of the balance between cell proliferation and cell death [8–10]. Therefore, the involvement of mitochondria in cell death, especially from the noncanonical view where mitochondria regulate cell death through manipulating the redox millieu will also be reviewed. Due to the fundamental regulatory role of mitochondria, disturbances of the integrity of their functions will result in cellular dysfunction leading to various pathological states or even death. Therefore, a good understanding of the basic mitochondrial biology will help in therapeutic design for better disease management. 

## 2. Mitochondrial Functions

### 2.1. Mitochondria Structure and Metabolism

#### 2.1.1. Mitochondria Structure

Mitochondria are essential to sustain life as around 98% of the oxygen that we breathe in is consumed by mitochondria for energy production. In order to understand the basic principles of mitochondrial bioenergetics, it is necessary to have a brief overview of its structure first. Mitochondria, as thought to have evolved from a bacterial progenitor and having their own mitochondrial DNA pool [11], are bounded by two membrane systems, including an outer and an inner membrane, and the space in between is referred to as the intermembrane space; however, the two layers of membranes occasionally come into contact with each other to form junctional complexes. The inner mitochondrial membrane, with multiple inward folding known as cristae which house membrane-bound mitochondrial enzymes, serves as the major barrier between mitochondria and cytoplasm since it is largely impermeable, thereby preventing small molecules and ionic species from entering the mitochondrial matrix [1].

#### 2.1.2. TCA Cycle and Electron Transport Chain

The tricarboxylic acid (TCA) cycle and the electron transport chain contribute to the key enzymatic components of the mitochondria. During the process of breaking down carbon substrates into acetyl CoA, reducing equivalents (NADH and FADH) are produced, which are then fed to the electron transport chain consisting of Complex I (NADH dehydrogenase), Complex II (succinate dehydrogenase), Complex III (ubiquinol cytochrome c reductase), and Complex IV (cytochrome c oxidase). Electrons move from the reducing equivalents to Complexes I and II, respectively, which are then passed onto ubisemiquinone for shuttling to Complex III followed by Complex IV through cytochrome c. In the meanwhile, an electrochemical proton gradient is generated when protons are transferred across the inner mitochondrial membrane into the intermembrane space coupled to the electron transfer at Complexes I, III, and IV. This is known as the proton-motive force, generating the mitochondrial transmembrane potential, which is usually about negative 150–180 mV as compared to the cytoplasm. The influx of the protons through the proton translocating F_1_F_0_-ATP synthase, driven by this force, is coupled to a chemical reaction that phosphorylates ADP to ATP [12]. An electrogenic transporter, adenine nucleotide translocase (ANT), then transports ATP out of the mitochondria to places where energy demanding cellular processes occur [1].

### 2.2. Mitochondria and Redox Homeostasis Maintenance

#### 2.2.1. Sources of Mitochondrial Reactive Radical Species

**Reactive Oxygen Species (ROS)**

Mitochondria are the major sources of free radical species production since unpaired electrons are generated in the process of oxidative phosphorylation. Partial reduction of molecular oxygen by the unpaired electrons leads to the production of superoxide anions (O_2_^∙−^) which are one of the reactive oxygen species (ROS) and readily converted to hydrogen peroxide (H_2_O_2_) by magnesium superoxide dismutase (MnSOD) residing in mitochondria matrix. H_2_O_2_ can be converted subsequently to the highly reactive oxygen species, hydroxyl radical (^∙^OH), through the Fenton reaction [7]. Unlike H_2_O_2_, O_2_^∙−^ does not diffuse that readily across the membrane and thus for O_2_^∙−^ produced in the matrix, the activity of MnSOD is critical to prevent the mitochondrial matrix components from oxidative damage.

Although, it is well known that ROS can be generated as byproducts of oxidative phosphorylation, the question as to the specific site(s) along the electron transport chain responsible for ROS generation has always been a hotly debated topic. It is traditionally believed that under physiological conditions, Complex I is the main site for mitochondrial ROS production, where O_2_^∙−^ is produced on the matrix side and rapidly dismutated to H_2_O_2_ [13, 14]. In addition, Complex III has also been reported as a site for O_2_^∙−^ production [15, 16]. It is demonstrated that under ischemic and apoptotic conditions, O_2_^∙−^ production is triggered at Complex III. This may happen through inhibition of Complex IV as well as over reduction of the electron transport chain in the event of mounting hypoxic stress [17]. In a more recent review [18], the relative contribution of each complex towards O_2_^∙−^ production has been clearly quantitated with Complexes I (producing O_2_^∙−^ to the matrix) and III (producing O_2_^∙−^ to both the matrix and intermembrane space) having the greatest maximum capacities while Complex II has normally negligible rates.

Another major source of ROS production in the cell is the NADPH oxidase (Nox) family proteins, which are enzyme complexes catalyzing the electron transfer from NADPH to molecular oxygen, generating O_2_^∙−^ and H_2_O_2_. The relative quantitative contribution of mitochondria and NADPH oxidase in cellular ROS production is expected to vary greatly from one cell type to another. In certain cells including the phagocytic neutrophils as well as nonphagocytic fibroblasts, vascular smooth muscle cells and endothelial cells, cellular ROS production is largely contributed by NADPH oxidase [18–20]. It has been recently reported that one of the Nox isoforms, Nox4, is expressed in the mitochondria in the rat renal cortex [21], cardiac myocytes [22, 23], and in the mitochondria-enriched heavy membrane fractions of the chronic myeloid leukemia cells that overexpress the antiapoptotic protein Bcl-2 (CEM/Bcl-2) [24]. Nox4 expression was shown to correlate with dihydroethidium staining for O_2_^∙−^. In Nox4-transgenic mice cysteine residues of mitochondrial proteins were also more oxidized [23]. However, there are no direct and specific measurements of Nox4 activity in the mitochondria up to date. Meanwhile, the cytoplasmic Nox4 may be involved in the activation of PKC*ε*, mitoK_ATP_ and modulation of thioredoxin 2 activity resulting in redox-sensitive upregulation of mitochondrial ROS production through the electron transport chain [25]. Apart from the electron transport chain and the NOX family, several other sites in the mitochondria have also been reported to generate O_2_^∙−^, including pyruvate dehydrogenase, *α*-ketaglutarate dehydrogenase [26], glycerol-3-phosphate dehydrogenase, and fatty acid *β*-oxidation [18].

Recently, new findings that supported the concept of mitochondrial O_2_^∙−^ flashes have gained much interest and revealed several previously unknown aspects of mitochondrial dynamics. Transient quantal O_2_^∙−^ flashes were observed in excitable cells such as muscle cells and neurons *in vivo* and they are associated with mitochondrial permeability transition pore (mPTP) opening, which presents a new facet in physiological ROS production [27–29].

**Reactive Nitrogen Species (RNS)**

Apart from ROS generation, there is evidence to suggest the expression of a mitochondrial specific nitric oxide synthase (NOS) leading to ^∙^NO production. It was first described in 1997 by Ghafourifar and Richter that ^∙^NO was generated from isolated mitochondria when they were loaded with calcium and mitochondrial potential dropped in some of the treated mitochondria [30]. Another group provided more direct evidence by showing images of calcium regulated ^∙^NO production within the mitochondria of permeabilized cells, through the use of the ^∙^NO-sensitive chromophore, DAF-2 [31].

The impact of ^∙^NO generation on mitochondrial functions remains controversial, largely depending on the amount of ^∙^NO that is produced as well as the conditions under which it is produced. It has been shown that mitochondrial respiration can be partially inhibited with even the modest levels of ^∙^NO causing an increase in mitochondrial ROS production. This also results in mitochondrial depolarization resulting in a decrease in mitochondrial calcium uptake [32]. The inhibitory effect of ^∙^NO on mitochondrial respiration has been shown to mainly result from inactivation of cytochrome c oxidase (Complex IV) [33–40], which is the rate-limiting step of the electron transport chain. Being a double-edged sword, ^∙^NO was demonstrated to play a role in mitochondrial biogenesis as well, which gets stimulated by ^∙^NO generation through a cGMP-dependent upregulation of PGC1*α* expression, which in turn increases the expression of mtTFA and NRF-1 resulting in an increased mitochondria biosynthesis observed in adipocytes and hepatocytes [41]. Another group also showed a physiological role for ^∙^NO generation in mitochondrial mass regulation where endothelial-^∙^NO-synthase- (eNOS-) deficient mice showed deficiencies in mitochondrial enzymes [42]. In addition, protective effect of ^∙^NO on mitochondria and cells is also observed against ischemia/reperfusion (I/R) injury (late preconditioning). It has been suggested that partial temporary inhibition of Complex I might be involved in this effect via inhibition of ROS burst in reperfusion. Furthermore, treatment with diet inorganic nitrates or administration of nitrites or S-nitroso-2-mercaptopropionyl glycine (SNO-MPG) confers cardioprotection against the injury via temporary S-nitrosylation of various cellular protein targets [43].

On the contrary, under some pathological stimuli, excessive production of ^∙^NO could result in severe tissue damage. Several groups have suggested that in the face of a high concentration of ^∙^NO with a relatively low oxygen concentration, respiration can be inhibited resulting in an increased O_2_^∙−^ production, which will react with the freely membrane diffusible ^∙^NO to form a much more reactive radical species, peroxynitrite (ONOO^−^). Peroxynitrite can also be formed through an alternative route via the reaction of nitroxyl anion (NO^−^) and molecular oxygen [44]. Nitroxyl anion can be derived from ^∙^NO through one electron reduction by the electron donors including cytochrome c [45] or ubiquinol [46]. Due to its ability to diffuse across the mitochondrial membranes, ONOO^−^ can result in oxidative damage of critical components throughout the mitochondria via oxidation, nitration, and/or nitrosation. For example, thiols present in Complex I can be oxidized leading to the formation of an S-nitrosothiol derivative and inactivation of the complex [47–49]. Complexes II and V were also shown to be inactivated by ONOO^−^ [34, 44, 48–53]. MnSOD, the matrix antioxidant enzyme, is also a target for nitration leading to a decrease in its enzymatic activity [54]. Adenine nucleotide translocase (ANT) [55], creatine kinase [56], nicotinamide nucleotide transhydrogenase [57], aconitase [58], and components of the pyridine nucleotide-dependent calcium release pathway [59] are all targets of ONOO^−^. Therefore, ONOO^−^ has profound effects on mitochondrial metabolism, calcium homeostasis, and the mitochondrial permeability transition pore [42]. In addition, ONOO^−^ has been shown to uncouple eNOS, leading to a switch from ^∙^NO to O_2_^∙−^ production and an increase in mitochondrial ROS levels as well [42, 60].

Of particular note, carbon monoxide (CO), an endogeneous gas produced by heme oxygenase (HO) that catalyzes heme degradation, is able to induce the production of ROS and RNS as well due to its high affinity for reduced transition metals such as Fe^2+^. For example, it binds to Complex IV and slows the terminal transfer rate of electrons to molecular O_2_ leading to enhanced O_2_^∙−^ production [61].

#### 2.2.2. Mitochondria in Redox Regulation

Since mitochondria are major sources for ROS production, it is not surprising that they are well equipped with antioxidant defenses, including a large pool of glutathione, glutathione peroxidase, glutathione reductase, MnSOD, catalase, and the thioredoxin system [1, 62].

Although excessive levels of ROS will lead to protein oxidation and lipid peroxidation causing damage to mitochondrial membrane, proteins, and DNA, especially when the mitochondrial DNA is not protected with associated histones, lower levels of ROS have been demonstrated to be essential signaling molecules [7, 63, 64]. A new concept is now emerging that mitochondrial ROS production is likely to be highly regulated as a part of physiological mitochondrial functions and the underlying molecular mechanisms are being gradually uncovered [7]. In this paper, a few mitochondrial proteins that act as redox regulators will be discussed as examples, including the antiapoptotic protein Bcl-2, cytochrome c oxidase (COX), and the small GTPase Rac1.

**Bcl-2 and Its Effect on Mitochondrial ROS Generation**

Bcl-2, one of the antiapoptotic members of the Bcl-2 family proteins residing on the outer mitochondrial membrane [65], is best known for its ability to inhibit apoptotic execution, that is, to form homo- and heterodimers to prevent the oligomerization of proapoptotic Bcl-2 family members, thus antagonizing the induction of mitochondrial outer membrane permeabilization (MOMP) [66]. However, recent evidence points to a new facet of Bcl-2 biology in redox regulation. The involvement of Bcl-2 in redox regulation was first demonstrated by Hockenbery et al. that Bcl-2 overexpression protected against ROS-induced apoptosis [67]. Soon after other studies also revealed the protective capacity of Bcl-2 against various ROS triggers [68–73]. However, Bcl-2 itself was later found out to possess no intrinsic antioxidant ability [74] implying that rather its overexpression indirectly induces an enhancement in antioxidant capacity of the cells when they undergo overt oxidative stress [66, 75] and this is supported by the findings that Bcl-2-mediated protection is associated with upregulation of the cellular enzymatic and nonenzymatic antioxidant defense machineries including the glutathione system, catalase, and NAD(P)H [74, 76–79]. More recently, a clearer picture of Bcl-2 with respect to its ability to regulate redox status is emerging, and a prooxidant role of Bcl-2 is established under normal physiological states [77, 80–84]. Based on this prooxidant property of Bcl-2, it implies that the enhanced antioxidant capacity that was observed with Bcl-2 overexpression could be an adaptive response to the chronic but mild oxidative intracellular milieu [70, 74, 76, 79, 83] and this serves as a first-line defense in the event of acute oxidative insults maintaining the ROS levels within a threshold optimal for cell survival [66, 75, 81, 85].

The underlying mechanisms on how Bcl-2 exerts its prooxidant activity, however, have not been fully elucidated. It was first hypothesized that the prooxidant milieu in Bcl-2 overexpressing mitochondria resulted from an altered dynamics of the oxidative phosphorylation. An increase in mitochondrial size and associated matrix content was observed with Bcl-2 overexpression and this indicated an increase in the number of electron donors and a subsequent increase in the chance of electrons leaking out of the electron transport chain to form O_2_^∙−^ [77, 86]. However, the exact mechanism linking Bcl-2 expression levels to mitochondrial size and matrix content was not addressed in the above-mentioned studies. More recently, our group has established the inherent ability of Bcl-2 to generate intramitochondrial O_2_^∙−^ by engaging mitochondrial respiration in tumor cells. An increased mitochondrial oxygen consumption rate and cytochrome c oxidase (COX or Complex IV) activity was observed in Bcl-2 overexpressing cells [81, 85, 87]. It is plausible that the increased mitochondrial respiration rate results in an increased electron flux across the electron transport chain and an increased probability of leakage of electrons onto molecular oxygen thus leading to an increase in the by-production of O_2_^∙−^. Indeed, either silencing of Bcl-2 with siRNA or functional inhibition of Bcl-2 with the BH3 mimetic, HA14-1, in those cells, reversed both the oxygen consumption rate as well as the O_2_^∙−^ levels [81]. This is further supported by the observation that mitochondrial respiratory rate and O_2_^∙−^ levels correlated with Bcl-2 expression levels across different tumor cell lines with various endogeneous Bcl-2 levels [85]. Of note, Bcl-2 overexpression promoted the mitochondrial localization of COX Va and Vb, which are nuclear encoded subunits of Complex IV, which could explain for the significantly increased Complex IV activity in these cells [85]; it has been previously shown that mitochondrial level of COX Vb correlated with the COX holoenzyme activity [88]. Of particular note, the increased O_2_^∙−^ release as induced by Bcl-2 overexpression might seem contradictory to what we mentioned in the earlier section that inhibition of ETC by ^∙^NO also results in O_2_^∙−^ release. However, the former occurs as a result of increased electron flux across the electron transport chain and an increased probability of leakage of electrons onto molecular oxygen while the latter is the result of inhibition of reduction step along the ETC leading to promotion of the reaction of oxygen with accumulated reductants [89].

More recently, our group has identified another functional player in Bcl-2-mediated prooxidant state, the small GTPase Rac1 [24]. Rac1 is known to be involved in the assembly and activation of NADPH oxidase complex leading to O_2_^∙−^ production. It was first discovered that introduction of the dominant negative mutant Rac1N17 neutralized the prooxidant activity of Bcl-2 [82]. Later on, a physical interaction was observed between these two proteins in the outer mitochondrial membrane of tumor cells which could be blocked with the BH3 mimetics and Bcl-2 BH3 domain peptides. The intramitochondrial O_2_^∙−^ production in Bcl-2 overexpressing cells was also reversed by BH3 peptides, which can also be achieved with silencing or functional inhibition of Rac1 [24].

These data provide evidence for the existence of functional complexes within the mitochondria involving Bcl-2, but the precise mechanism of interaction and how disruption of these interaction(s) could impact cell fate remains to be elucidated. Of course, apart from the above-mentioned proteins, there are many others that act as mitochondrial redox regulators including the master transcription factor for cellular antioxidant defense machinery, the transcription factor NF-E2-related factor 2 (Nrf2), which gets activated under cellular oxidative stress conditions such as GSH depletion, ^∙^NO, and nitrosative stress [90–92]. Interestingly, a recent paper provided the first evidence that activation of Nrf2 can upregulate Bcl-2 as well [93].

### 2.3. Mitochondria and Cell Fate Regulation

Since mitochondria are fundamental energy generators, severe damage to mitochondria will inevitably cause disorders in cellular functions. Once ionic gradients and intracellular osmolarity cannot be maintained, cells will swell and die through a death process known as necrosis [94]. However, for some immortalized cells, they survive reasonably well on ATP generated from glycolysis even when mitochondrial respiration is completely inhibited [1, 95, 96]. Apart from dying passively when the ATP supply fails, cells can also actively undergo a “suicide” program through the mitochondria-mediated apoptotic pathway upon compromise in the mitochondrial outer and/or inner membrane permeability [97]. Furthermore, there is another form of cell death named autophagy, which degrades cellular organelles and proteins promoting either survival or death depending on the stress conditions. Various studies have indicated the involvement of ROS and mitochondria in autophagic regulation [98, 99]; however, due to space constraints, it is not covered in this review.

#### 2.3.1. Apoptosis

For the past two decade or so, mitochondria have been extensively studied for its essential role in defining the balance between cell life and death. It was first demonstrated in 1994, that cytochrome c, once it is released from the intermembrane space of the mitochondria, can initiate an enzyme cascade of cellular self-destruction. Other death amplification factors, such as the inhibitors of apoptosis (the IAP family which prevent accidental caspase activation presumably), smac or *diablo* (which inhibits IAPs to permit the apoptotic cascade to proceed), procaspase-9, and AIF (the flavoprotein apoptosis inducing factor) are also released to participate in the death execution pathway [97].

#### 2.3.2. Redox Status in Cell Fate Decision

Although overwhelming amount of ROS are definitely detrimental to the cells, emerging evidence has demonstrated that when they are present in nonlethal concentrations, they can function as proliferative and/or survival signals [8]. A mild increase in the O_2_^∙−^ has been shown to confer survival advantage to the tumor cells under apoptotic triggers [8, 10, 100–103]. Furthermore, it has been demonstrated by our group that cell fate is tightly regulated as a function of the ratio of O_2_^∙−^ and H_2_O_2_. A tilt in the balance of the two reactive oxygen species towards O_2_^∙−^ leads to survival signaling, while the reverse sensitizes cells to apoptotic triggers [8–10]. Corroborating the survival advantage of a mild prooxidant status, the antiapoptotic activity of Bcl-2 can be contributed to its noncanonical ability to induce intramitochondrial O_2_^∙−^ production. Indeed, Bcl-2 BH3 peptides, which reversed the prooxidant state of Bcl-2-overexpressing tumor cells, sensitized those cells to drug-induced apoptosis. Similarly, silencing or pharmacological inhibition of Rac1 also compromised Bcl-2-induced increase in intramitochondrial O_2_^∙−^ levels leading to sensitization of those tumor cells to apoptotic triggers [24]. 

## 3. Mitochondrial Dysfunction in Pathology and Therapeutic Targeting

Mitochondria, as one of the major ROS producers within the cell, have been rendered susceptible to oxidative damage when the antioxidant defense machinery fails to meet their ROS scavenging tasks; therefore, they are implicated in the pathology of various diseases including neurodegenerative diseases, diabetes, cardiovascular diseases, and cancer [104–114].

### 3.1. Neurodegenerative Diseases

ROS-mediated mitochondrial dysfunctions and apoptosis have been demonstrated as causal factors in the pathology of several neurodegenerative diseases including Parkinson's disease (PD), Alzheimer's disease (AD), and Amyotrophic lateral sclerosis (ALS). Oxidative damage, as indicated by malondialdehyde (MDA) and 4-hydroxynonenal (4-HNE), have been identified in patients diagnosed with PD (substantia nigra), AD (hippocampus and cortex) as well as ALS (spinal fluid) [115–118]. The levels of Iron are also found to be elevated in the substantia nigra of patients with PD, which could serve as a catalyst for the Fenton's reaction in producing hydroxyl radicals [119–121], whereas the activities of antioxidant defense enzymes such as glutathione peroxidase (GPx) and reductase (GR), superoxide dismutase (SOD) and catalase (CAT) are reduced in the affected brain regions of patients with AD [117, 122, 123]. Similarly, patients with PD demonstrate diminished levels of GSH in the dopaminergic neurons of substantia nigra [118, 119, 124].

Transgenic animals that develop neurodegenerative diseases have been utilized as models to clarify the roles of oxidative stress in the pathogenesis of these diseases. Transgenic mice that harbor an ALS-linked mutant CuZnSOD gene show progressive accumulation of 8-OHdG, one of the best markers for oxidative DNA damage, in ventral horn neurons. The immunoreactivity for this marker indicates the existence of oxidative damage to mitochondrial DNA in spinal motoneurons starting from very early stage of the disease, and probably contributing to the subsequent motoneuron death [125]. In another model of GPx deficient mice, administration of N-methyl-4-phenyl-1,2,3,6-tetrahydropyridine (MPTP) led to enhanced toxicity to dopaminergic neurons indicating the involvement of ROS in the early pathogenesis of PD [126]. Triple-transgenic mice that mimic AD progression in humans also demonstrate reduced levels of GSH and vitamin E as well as increased extent of lipid peroxidation during the stage of Amyloid *β* (A**β**) oligomerization before the onset of A**β** plaques and neurofibrillary tangles [127].

Since oxidative stress resulting from depletion of the cellular antioxidant glutathione occured during the early stages of neurodegeneration, a lot of effort has been dedicated to study the effects of antioxidants in combating oxidative stress and preserving the integrity of mitochondria for the treatment of neurodegenerative diseases. Several examples of antioxidants that have been demonstrated to attenuate disease progression will be discussed below.

**Curcumin**

Curcumin is a polyphenol that belongs to the ginger family (Zingiberaceae). Curcumin treatment of dopaminergic neuronal cells and mice restores GSH pools, which protects against oxidative stress and preserves mitochondrial complex I activity, thereby suggesting its therapeutic benefits in the treatment of PD [128]. Protective effects of curcumin have also been shown against MPP (+)-induced cytotoxicity and apoptosis by upregulation of Bcl-2 expression and restoring mitochondrial membrane potential [129]. In addition, tetrahydrocurcumin (THC), a metabolite of curcumin, has been shown to protect against A**β**-induced ROS burst, preserve mitochondrial membrane potential, and prevent caspase activation in rat primary hippocampal cultures [130].

Apart from oxidative stress, nitrosative stress, largely mediated by reactive nitrogen species (RNS) such as OONO^−^, is also crucial in PD development by inducing mitochondrial dysfunction through inhibition of brain mitochondrial complex I activity, decrease of mitochondrial membrane potential, and compromise of mitochondrial integrity. The glutamoyl diester bioconjugate of curcumin has been shown to restore Complex I activity and protect against protein nitration [131].

**Epigallocatechin-3-Gallate (EGCG)**

EGCG is the most abundant polyphenol found in green tea. Apart from its iron-chelating property, the antioxidant capacity of EGCG has been demonstrated at the level of mitochondria where it not only enhances the activities of both TCA cycle enzymes and ETC complexes but also upregulates the antioxidant system in aged brain [132]. EGCG has also been shown to increase the activity of SOD and catalase in mice striatum [133]. The molecular mechanism underlying the upregulation of the antioxidant defense machinery is due to the ability of EGCG to induce the activation of Nrf2, a master transcription factor for antioxidant and phase II detoxifying enzymes [134].

Although these molecules demonstrate protective effects in animal models of the neurodegenerative diseases, their beneficial effects in humans have not been clearly demonstrated in clinical trials, conceivably due to the difficulties in penetrating the blood-brain barrier; therefore a lot of effort is currently being spent in developing better delivery systems for specific targeting, especially to mitochondria, where their pharmacological activity is mostly required to increase the therapeutic efficacy [135–138].

### 3.2. Diabetes, Diabetic Complications, and Cardiovascular Diseases

Despite the fact that diabetes mellitus (DM) is a heterogeneous, multifactorial and chronic disease, it can be stated without doubt that DM is linked to acute and continuous overproduction of ROS and characterized by mitochondrial impairment. DM is also marked with chronic inflammation that further weakens intracellular antioxidant defense. Reduced levels of antioxidants such as GSH, vitamins C and E are observed in diabetic patients [139, 140]. Dysfunction of mitochondrial complex I and subsequent increase in ROS production together with decreases in antioxidant levels and membrane potential have been reported in diabetic patients [141]. Risk factors such as aging, obesity, and unhealthy diet contribute to an oxidative environment which impairs insulin signaling and mitochondrial function leading to diabetes development, and the resulting hyperglycemia in turn contributes to the maintenance and progression of the overall oxidative stress through mechanisms such as glycation of antioxidant enzymes [111] and overproduction of O_2_^−^ by the mitochondrial ETC, which in turn activate a variety of proinflammatory signals [111, 142–144].

Malfunctioning of mitochondrial oxidative phosphorylation has been considered as one of the main culprits in the development of diabetic complications as well, such as renal dysfunction [145]. In addition, it has been reported that the activities and expression of antioxidant enzymes are decreased in diabetic microvascular disease [146, 147] and a specific polymorphic MnSOD gene is correlated with diabetic nephropathy development [148]. Protective effects of catalase overexpression have also been demonstrated in the experimental models of type 2 diabetic nephropathy, thereby implicating H_2_O_2_ [149]. Majority of the type 2 diabetic patients with insulin resistance also exhibit a significantly higher risk of developing cardiovascular disease (CVD) [150]. Human atherosclerotic samples show higher extent of mitochondrial DNA damage, which correlates with greater ROS production. In apoE-null mice, mitochondrial damage has been shown to precede the development of atherosclerosis. In the same model, heterozygous deficiency of MnSOD also increases vascular mitochondrial dysfunction [151].

Since oxidative stress has been considered as one of the main contributing factors for the onset and progression of diabetes, diabetic complications, and CVD, the need to eradicate ROS especially from mitochondria is of great therapeutic importance [152]. Although classical antioxidants such as vitamins C and E do not show significant improvement in disease conditions [153], recent reports show that a subgroup of type 2 diabetic patients with haptoglobin (Hp) 2-2 genotype can benefit from *vitamin E* supplementation suggesting that tailored treatment regimens for different subgroups of patients could be more favorable [154, 155]. *MitoQ* is an antioxidant that is selectively targeted to and accumulates in mitochondria due to its covalent attachment to the lipophilic triphenylphosphonium cation [156]. MitoQ administration to Ins2(+/) (AkitaJ) mice improves the tubular and glomerular function and reduces urinary albumin levels and interstitial fibrosis implicating their therapeutic benefits in treating diabetic nephropathy [157].

### 3.3. Cancer

#### 3.3.1. Prooxidant Theory of Carcinogenesis

We have discussed in the previous Section 2.3.2 how redox status can affect cell fate decision and how some mitochondrial proteins can act as redox mediators. Over the past decade, our group has been working on the underlying mechanisms and translational relevance of redox signaling in the context of carcinogenesis. We have established that cell fate is tightly regulated as a function of the ratio of O_2_^∙−^ and H_2_O_2_. A tilt in the balance of the two reactive oxygen species towards O_2_^∙−^ leads to survival signaling while the reverse sensitizes cells to apoptotic triggers [8–10].

#### 3.3.2. Mitochondria as Therapeutic Targets in Cancer

Since mitochondria are key regulators for energy metabolism, ROS production and cell fate [158], targeting mitochondria to elicit cell death would therefore be a good strategy in cancer therapeutics especially in those cancer cells where upstream apoptotic signaling is malfunctional [159]. In addition, the bioenergetic differences between nontransformed and cancer cells confer the selectivity and specificity in targeting small molecule agents to the mitochondria of desired cancer cells. For example, lipophilic cations preferentially accumulate in the mitochondrial matrix of cancer cells due to their increased mitochondrial membrane potential resulting from increased glycolytic rates as compared to their normal counterparts [160, 161]. In addition, the addiction of cancer cells to glycolysis for ATP supply, described as the Warburg effect, also renders them more susceptible to apoptotic induction when their cellular bioenergetic pathways are intervened [161].

**Direct Targeting of Mitochondrial ETC**

Since mitochondrial ETC is essential in energy production and ROS generation, there are a plethora of agents that target ETC directly for cancer therapeutics [106]. Rotenone is a naturally derived hydrophobic pesticide, which binds and inactivates mitochondrial Complex I irreversibly leading to a blockage of oxidative phosphorylation and an increase in ROS generation [162, 163] and finally apoptotic induction [164–167]. Therapeutic benefits of Rotenone have been demonstrated in human breast cancer cells [168], neuroblastomas [169], promyelocytic leukemias [170], and human B-cell lymphomas [167]. Tamoxifen and estradiol have also been reported to act on the flavin mononucleotide site of complex I leading to mitochondrial failure independent of estrogen receptors [171], and particularly in MCF-7 breast cancer cells tamoxifen is able to induce an increase in ROS levels, a decrease in mitochondrial membrane potential and release of cytochrome c [172]. 3-nitropropionic acid is a toxin found in fungi and plants that can bind covalently to complex II [173–175] and its toxicity to tumor cells is linked to cellular energy depletion and oxidative stress due to the generation of O_2_^−^, H_2_O_2_, and OONO^−^ [176, 177]. In addition, an analog of vitamin E, *α*-tocopheryl succinate (*α*-TOS) is able to interfere with the ubiquinone binding site on Complex II [178] leading to cell cycle arrest and apoptosis in a host of established cancer cell lines of different origin as well as in *in vivo* experimental animal models [179–185]. Although *α*-TOS has been shown to be largely nontoxic to normal tissues [183], its efficiency has not yet been tested in human cancer patients due to the difficulties in administration [186]. Antimycin A is a secondary metabolite produced by *Streptomyces kitazawensis *[187] which binds to the Qi site of Complex III [188, 189] leading to collapse of the proton gradient [188], ROS production, and apoptosis [190]. Fenretinide is a synthetic analog of retinoic acid which is able to downregulate Complex IV subunit III mRNA levels leading to a decrease in Complex IV activity [191]. Fenretinide induces apoptosis through elevated ROS production, cytochrome c release, and induction of mitochondrial permeability transition [192, 193], which could be prevented by the addition of antioxidants [194, 195]. It is likely that fenretinide inhibits at least one of the complexes along the ETC, although the exact prooxidant mechanisms are yet to be elucidated [194, 196]. *In vivo* therapeutic benefits of fenretinide have been demonstrated in both carcinogen-induced or xenograft animal models [197].

Direct targeting of mitochondrial ETC increases ROS production from the mitochondria of cancer cells which results in increased susceptibility of those glycolytic addicted cells to apoptotic induction. However, the critical points to be taken into consideration when using mitochondrial respiration “poisons” are their *in vivo* toxicity and therapeutic indexes. Almost half of the studies as discussed above failed to actually demonstrate nontoxicity of the agents to nontransformed cells *in vivo* except for *α*-TOS and fenretinide.

**Direct Targeting of Bcl-2 Family Proteins**

Apart from targeting the mitochondrial ETC, there is another group of proteins that are of particular interest due to their regulatory roles in apoptosis, which is the Bcl-2 family. We have discussed in Sections 2.2.2 and 2.3.2 that the ratio between the pro- and antiapoptotic members of Bcl-2 family is critical in cell fate decision. In addition, Bcl-2, an antiapoptotic member of the family and a resident protein of mitochondria, is able to modulate redox status which could be utilized in cancer therapeutics as well. Promising therapeutic strategies that aim at overcoming the problem of Bcl-2 overexpression (which happens in a number of cancers) including Bcl-2 antisense and BH3 mimetics have been recently reviewed by our group [66]. Furthermore, treatment of membrane active segments of the proapoptotic member Bax can also induce apoptosis in tumor cells [198].

**Indirect Targeting of Mitochondrial Apoptotic Pathway**

There are another group of drugs that do not target mitochondria directly but rather modulate mitochondrial proteins and/or induce ROS production leading to induction of intrinsic mitochondrial apoptotic pathway in cancer cells. These include clinically used chemotherapies such as irinotecan, topotecan [199], etoposide [200], vinblastine [201], and arsenic trioxide [202], which induce mitochondrial apoptotic pathway as well as those currently undergoing clinical evaluation such as betulinic acid [203], curcumin [204], camptothecin derivatives [199], and triapine [205], where betulinic acid and triapine-induced apoptosis has been attributed to ROS production.

## 4. Concluding Remarks

Mitochondria are essential regulators of cellular energy metabolism, redox homeostasis, and cell fate decision, and their dysfunction inevitably leads to various pathological states including neurodegenerative diseases, diabetes, cardiovascular diseases, and cancer as briefly discussed in this review. Oxidative stress is the underlying causal factor in majority if not all of the diseases listed above; therefore, therapeutic strategies that aim at manipulating the redox metabolism represent promising options which have been and will still be at the center stage of targeted drug development.
